# Different CMV-specific effector T cell subtypes are associated with age, CMV serostatus, and increased systolic blood pressure

**DOI:** 10.1186/s12979-025-00523-x

**Published:** 2025-07-03

**Authors:** Lennart M. Roesner, Berislav Bošnjak, Stephan Traidl, Jochen Huehn, Reinhold Förster, Thomas Werfel

**Affiliations:** 1 Department of Dermatology and Allergy, Hannover Medical School (MHH), Carl-Neuberg-Str. 1, 30625 Hannover, Germany; 2 Institute of Immunology, Hannover Medical School (MHH), Hannover, Germany; 3 Department Experimental Immunology, Helmholtz Centre for Infection Research (HZI), Braunschweig, Germany; 4 Cluster of Excellence RESIST (EXC2155), Hannover Medical School (MHH), Hannover, Germany

## Abstract

**Background:**

Cytomegalovirus (CMV) infection is one of the most common infections in humans, and CMV antigens are the major drivers of repetitive T-cell stimulation as a part of a well-adapted immune response in immunocompetent individuals. With higher age, the recurrent clonal expansion of CMV-specific T cells results in high frequencies of CMV-specific effector T cells. Further on, CMV seropositivity has been linked to an increased risk of developing cardiovascular diseases (CVD). Here we investigated the frequency and phenotype of CMV-specific T cells in the circulation of a population cohort of 650 individuals focusing on the age group over 60 years. Circulating immune cells of individuals carrying the HLA-A*02 allele were investigated (*n* = 302) applying MHC class I tetramers.

**Results:**

We add to previous knowledge by showing that the frequency of CMVpp65-specific CD8^+^ T cells is associated with the total percentage and absolute counts of CD8^+^ and CD4^+^CD8^+^ double-positive T cells within leukocytes, and further with systolic blood pressure (SBP) and history of CVD. An investigation into the differentiation status of CMV-specific T cells revealed an association of higher age and increased frequencies of both T_EM_ and CD27-expressing T_EMRA_ cells. In contrast, higher CMV-IgG titers were found to be associated with T_EM_ and CD27^−^ T_EMRA_ cell frequencies. SBP significantly correlated with CMV-specific effector CD8^+^ T cells, which was mostly reflected by CD27^−^ T_EMRA_ cells.

**Conclusions:**

Within the circulating CMV-specific T cell population, different effector T-cell subtypes were associated with age, serostatus and SBP. This suggests that it is not age or infection per se that render CMV-positive individuals susceptible to CVD, but rather the cellular immune response to CMV. Detailed immunophenotyping may identify individuals whose immune systems are strongly influenced by the response to CMV, leading to health consequences and impairing healthy aging.

**Supplementary Information:**

The online version contains supplementary material available at 10.1186/s12979-025-00523-x.

## Introduction

Aging is a progressive biological process in most multicellular organisms that leads to the gradual loss of body and organ functions and ultimately to biological death. Aging of the immune system is known as immunosenescence, a term that encompasses innate and adaptive immune dysfunction leading to increased infection and tumor susceptibility, and diminished response to vaccination [1]. One of the main characteristics of immunosenescence is misbalance of T cell immune responses induced by thymic involution and consequent reduction in the T cell receptor diversity, changes in cellular metabolism, and epigenetic alternations [2].

Particularly strong impact on immunosenescence have infections with herpesviruses, such as cytomegalovirus (CMV). CMV manages to establish a lifelong latent infection from which it sporadically reactivates and leads to expansion of CMV-specific T cells in a process described as a memory inflation [3]. In CMV-positive elderly individuals, CMV-specific CD8 T cells can account for up to a quarter of the total CD8 T cell population [4]. The accumulation of CMV-specific CD8 T cells exhausts the immune system over time and accelerates immunosenescence [5]. Hence, it is not surprising that CMV-seropositivity was correlated to a reduced ability to respond to novel threats in the elderly [6, 7], but not in young adults [8]. Therefore, latent CMV infection has the potential to influence the course of further infections, due to the broader reshaping of cytotoxic lymphocyte populations. On the other hand, CMV immunity has been linked to arterial stiffness [9, 10], increased systolic blood pressure [11], and an increased risk towards cardiovascular diseases (CVD) [12–14]. Earlier studies addressed the phenotype of CMV-reactive T cells, described the predominant T_EMRA_ phenotype, which reflects the contiuous antigen recognition due to constant reactivation of the virus, and showed an impact on the overall T cell composition [15, 16]. Not surprisingly, CMV infection is correlated with an increased risk of frailty and mortality [17–20].

In a meta-analysis that included data from more than 34,000 individuals, it was calculated that CMV seropositivity increased the relative risk of developing CVD by 22% [21]. However, several subsequent clinical studies did not confirm the association between CVD and serum CMV antibody levels [22–24]. Demographic characteristics in CMV seropositivity [25] or the discrepancies in the age of study participants only partially explain reported inconsistencies in the association of CMV infection and CVD. On the other hand, the CMV-specific CD8 T-cell responses correlate to arterial stiffness in hypertension patients [9, 10]. Given the involvement of senescent T cells in cardiovascular diseases [26–28], CMV infection-driven memory inflation may potentiate CVD risk by increasing the number of immunosenescent T cells. Of note, CMV-specific CD8 T-cell responses correlated to CMV antibody titers in some [29–31] but not all studies [32–34]. Hence, it is possible that the CMV-specific T cells would be a better predictive risk factor for CVD than CMV-specific antibodies.

To investigate the frequency and phenotype of CMV-specific T cells in the circulation and their impact on CVD, we used the RESIST senior individuals (SI) cohort [35]. This population-based cohort of 650 citizens focused on the age group of 60–100 years was set up between 2019 and 2023 to investigate age-related changes in the immune system. Given the fact that the extent of CMV-specific CD8 T cell responses depends on the HLA haplotype [36], we focused exclusively on HLA*A02 + donors, as HLA*A02 was found to be the most frequent HLA class I allele in a representative German population [37]. Here, we report on frequency of CMV-specific T cells from fresh blood and their differentiation status with regard to age, sex, antibody titer, blood pressure, and patient-reported history of cardiovascular diseases.

## Methods

### Study subjects

This study was performed within the RESIST senior individuals (SI) cohort, which was set up as a general population cohort to describe and investigate age-related differences and changes in the human immune system. 550 individuals aged over 60 years as well as 100 young adults aged 20 to 40 years were randomly selected by the local residents’ registration office in Hannover, Germany, and invited to participate from 2019 to 2023. It is important to note that the use of systemic anti-inflammatory or immunosuppressive medications and previous organ-transplants were defined as exclusion criteria, with the objective of concentrating the study on age-related changes [35].

### Antibody titer testing

Blood was drawn without anticoagulant for serum sampling and with EDTA for PBMC isolation. Serum samples were tested in the routine diagnostic of the clinical virology lab at MHH. Serology was performed on the Architect System from Abbott Diagnostics (Abbott GmbH & Co. KG, Wiesbaden, Germany) using the CMV IgG assay for detection of IgG antibodies against CMV. The detection of IgG antibodies for VZV and HSV was done on the Liaison XL analyzer (DiaSorin S.p.A. Saluggia (VC), Italy) using the LIAISON^®^ VZV IgG and the LIAISON^®^ HSV-1/2 IgG assay. While this assay is a semi-quantitiative assay, it cannot be excluded that among the participants with very low values are also CMV-seronegative donors.

### Immune cell count

Whole fresh blood was subjected to FACS Lysing Solution (#349202, BD Biosciences, Franklin Lakes, NJ, USA) and subsequently the 6-color TBNK Reagent with TruCount tubes (#337166, BD Biosciences) according to the manufacturer’s protocol. Fluorescence signals and TruCount beads were acquired on a 3-laser CytoFLEX using Cytexpert software 2.3 (Beckman Coulter, Brea, CA, USA). Instrument quality control and standardization were performed daily using CytoFLEX Daily QC Fluorospheres (Beckman Coulter #B53230).

### Analysis of CMV-specific T cells

The following analyzes were performed without prior freezing/thawing of cells directly after obtaining the blood samples: HLA-A2 status was determined by antibody staining (clone BB7.2, BD Biosciences) on CD45^+^ cells (clone HI30, BioLegend, San Diego, CA, USA) in fresh blood after subjecting to FACS Lysing Solution (BD Biosciences) by means of flow cytometry. PBMC were isolated by density-gradient centrifugation on Ficoll (Pan Biotech, Aidenbach, Germany) and subjected to subsequent analyses. 1 × 10^6^ cells were incubated with MHC-I-tetramers, consisting of tetramerized and PE-labelled HLA-A*02:01 monomers carrying the NLVPMVATV peptide CMVpp65_495 − 503_ (1002-07 ImmunAware, Horsholm, Denmark; IEDB#: 44920) at room temperature for 30 min. Subsequently, anti-CD8-BV510 (clone SK1, Biolegend 344732), anti-CD27-APC (clone O323, Biolegend 302810), anti-CD45RO-PacificBlue (clone UCHL1, Biolegend 304216) and a viability dye (ThermoFisher/Invitrogen/eBioscience fixable viability dye eFluor780) were added and incubated for 30 min at 4 °C. After washing, cells were analyzed on aforementioned 3-laser CytoFLEX using Cytexpert software 2.3. Data were analyzed with the Software Kaluza V1.3, BeckmanCoulter. In mean 1722 (SD = 625) CD45 + lymphocytes were analyzed per participant. For subgrouping of CMVpp65 tetramer + T cells, only measurements with at least ten clearly positive events were considered.

### Statistical analyses

We used GraphPad Prism (Version 5.0.0, GraphPad, La Jolla, USA) and R (version 4.1.2) with the gt-summary package (version 1.7.0) for statistical analyses using tests as listed under each figure. The Mann-Whitney U test was applied when comparing two groups. The Kruskal-Wallis test with Dunn´s multiple comparison test was used for comparing more than two independent samples. Spearman’s rank correlation was applied to measure the strength and direction of association between two ranked variables. A generalized linear model (GLM) with a Gaussian distribution and identity link function was used to assess the association between CMV-specific T cell populations and clinical covariates. The model included age (continuous), sex (binary), body mass index (continuous), and grouped frequencies of CMV-specific T cells (categorical) as fixed effects. For visualization, the plot_model() function in the sjPlot R package was used. Results with a *p*-value < 0.05 were considered statistically significant (*p < 0.05; **p < 0.01; ***p < 0.001).

### Sex as a biological variable

Our study examined male and female participants, and similar findings are reported for both sexes.

## Results

### Stratification of participants of the RESIST SI cohort

**Fig. 1:**
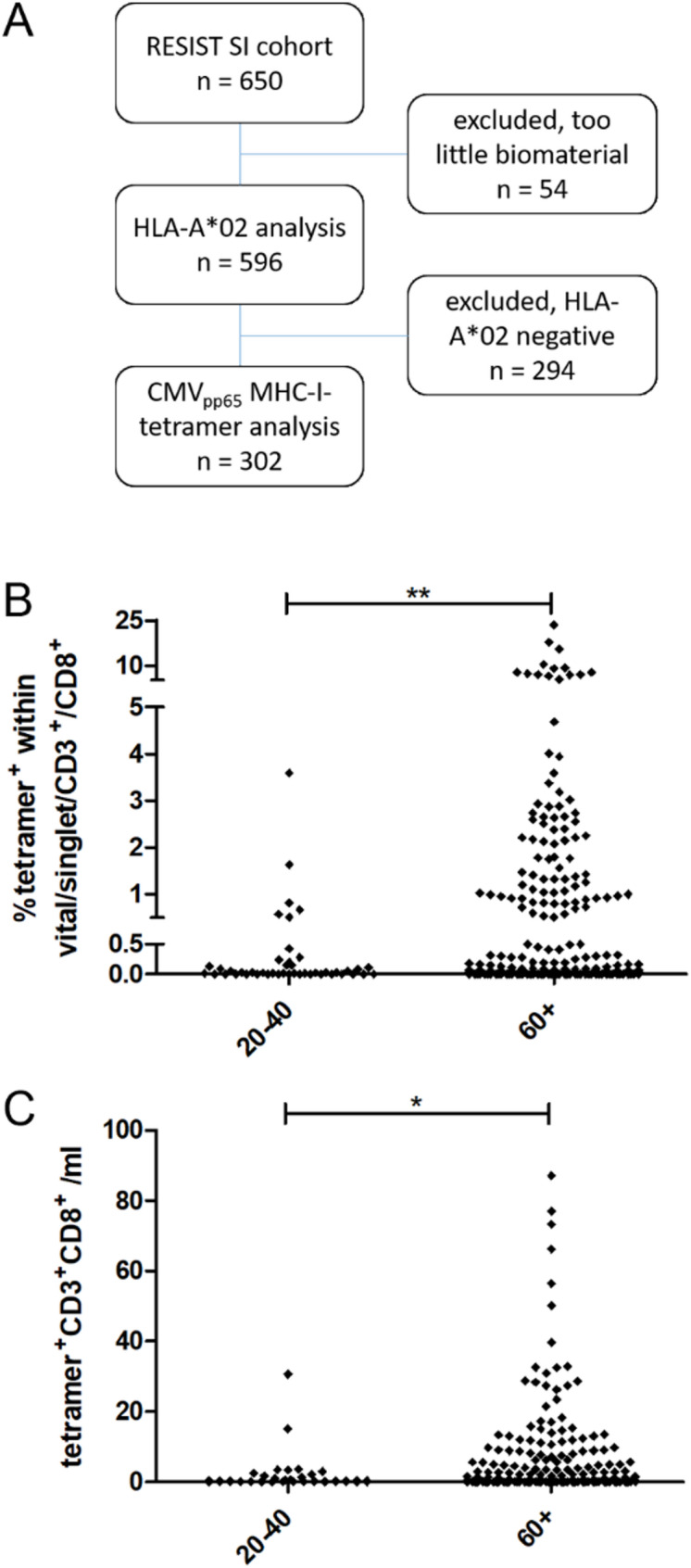
Study outline and Frequency of CMV-specific T cells. **A**, Study outline. From the 650 healthy volunteers of the RESIST senior individuals (SI) cohort, a total of 302 individuals was analyzed by means of CMVpp65_495 − 503_ MHC-I-tetramer staining. **B**, Frequency of CMVpp65_495 − 503_ MHC-I-tetramer^+^ T cells comparing the age groups of 20–40 and 60 + year old healthy volunteers. **C**, Absolute cell counts of CMVpp65_495 − 503_ MHC-I-tetramer^+^ T cells comparing the age groups of 20–40 and 60 + year old healthy volunteers

In total, 650 individuals, 550 of whom were aged 60 or over, were recruited in the RESIST SI cohort and followed a structured study visit as depicted earlier [35]. During the first study visit, we analyzed HLA-A*02 expression on blood cells using flow cytometry if there was enough material available for the follow-up examinations on CMV-specific T cells. Of the 596 analyzed samples, 51% (*n* = 302) stained positive for HLA-A*02 (Fig. 1A) and were used to analyze the frequency of CMV-specific T cells within the T cell fraction using MHC-I tetramers carrying the CMVpp65 peptide epitope NLVPMVATV. Absolute counts of lymphocytes were 1930 (627) cells/µl in mean (SD) in the 20–40 year-olds, and 1685 (622) cells/µl in the 60 + year-olds. Overall, the CD4/CD8 ratio was 1.77, with 1.70 in the 20–40 year-olds and 2.33 in the 60 + year-olds. Frequencies as well as absolute counts of CMV-specific T cells comparing young adults (20–40 years-old) and elderly (60 years or older) individuals are given in Fig. 1B-C and supplemental Fig. 1. Confirming earlier reports, the frequency was increased in the elderlies, while no significant differences were to be seen when comparing male and female participants (Supplemental Fig. 2).

### The CMV-specific T cells frequency correlates with blood T cell frequencies and absolute cell counts

**Fig. 2:**
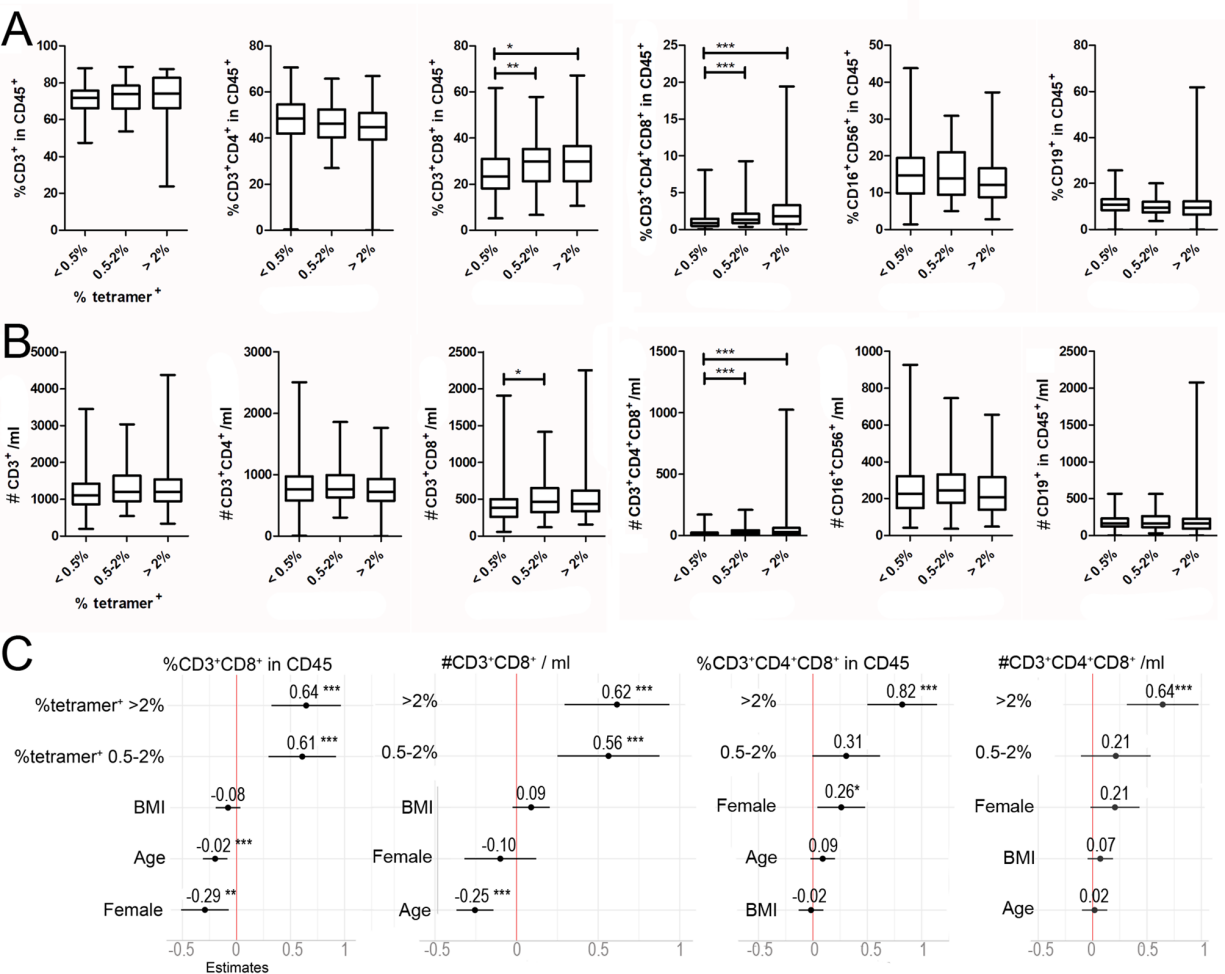
Association of the frequency of CMV-specific T cells and total leukocyte frequencies and counts. Subjects were grouped according to the frequency of CMVpp65_495 − 503_ MHC-I-tetramer^+^ T cells within the vital CD3 + CD8 + fraction as indicated into < 0.5%, 0.5-2%, and > 2%. **A**, Frequencies of T cells (CD3^+^), CD3^+^CD4^+^, CD3^+^CD8^+^, CD3^+^CD4^+^CD8^+^ double positive T cells, NK cells (CD16^+^CD56^+^), and B cells (CD19^+^) as determined by TruCount flow cytometry in fresh blood. **B**, Absolute counts of T cells (CD3^+^), CD3^+^CD4^+^, and CD3^+^CD8^+^, CD3^+^CD4^+^CD8^+^ double positive T cells, NK cells (CD16^+^CD56^+^), and B cells (CD19^+^) as determined by TruCount flow cytometry in fresh blood. **A**, **B**, Kruskal-Wallis test with Dunn´s multiple comparison testing. **C**, Forest plots of generalized linear models. From left to right, total frequencies of CD3^+^CD8^+^, total counts of CD3^+^CD8^+^, frequencies of CD3^+^CD4^+^CD8^+^ and total counts of CD3^+^CD4^+^CD8^+^ were tested against the groups of tetramer + frequencies, body mass index (BMI), age, and female sex. *p < 0.05; **p < 0.01; ***p < 0.001

In line with previous reports [32, 38], CMVpp65-specific T cells varied strongly between individuals, with frequencies often exceeding 0.5% of total CD8 T cells. To investigate whether expansion of CMV-specific T cells affects the overall leukocyte composition within the blood, we subgrouped the cohort according to the frequency of CMVpp65 MHC-I-tetramer^+^ cells within the CD8 fraction to a low (< 0.5%), a medium (0.5-2%), and a high (> 2%) group. Individuals of the low group had significantly less total CD8^+^ and CD4^+^CD8^+^ double-positive T cell frequencies compared to the medium and the high group (Fig. 2A). This was mostly reflected by absolute counts of CD8^+^ and CD4^+^CD8^+^ T cells (Fig. 2B). Spearman´s correlation analysis confirmed that the frequency of CMVpp65 MHC-I-tetramer^+^ cells was positively associated with the frequency of total CD3^+^/CD8^+^ (*p* < 0.0001), the absolute count of CD3^+^/CD8^+^ (*p* = 0.003), as well as the frequency and absolute count of CD3^+^/CD4^+^/CD8^+^ (both *p* < 0.0001). Since also for the factors age, sex, and body mass index an impact on the composition of the blood count has been described, we applied generalized mixed models to exclude confounding effects: Fig. 2C shows that CMV-specific T-cell frequency is responsible for the effects, while age and sex partially confound the different parameters and blur the effects. Our data are, therefore, in agreement with previous findings indicating that CMV infection is one of the most influential non-genetic factors that reshape the cellular composition of the immune system [39].

### Higher frequencies of CMV-specific CD45RO^+^/CD27^−^ and CD45RO^−^/CD27^lo − int^ T cell populations in elderlies

**Fig. 3:**
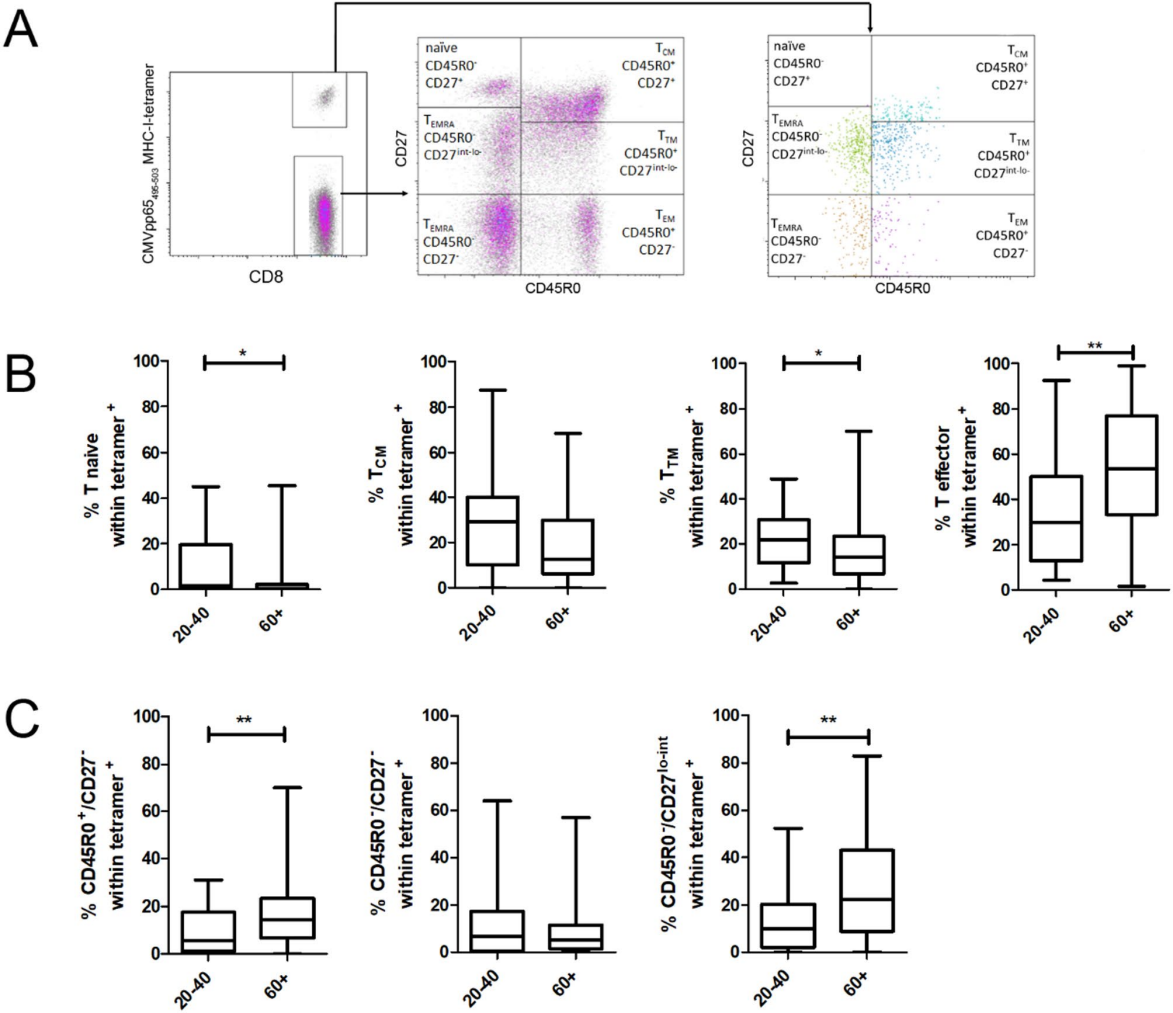
Frequency and differentiation status of CMV-specific T cells with regard to age. **A**, Flow cytometry gating scheme discriminating the differentiation status of the CMV-specific T cells. Vital, singlet CD8^+^ lymphocytes of HLA-A*02 positive individuals were incubated with PE-labelled CMVpp65_495 − 503_ MHC-I-tetramers and antibodies to CD27 and CD45RO to subtype CMV-specific T cells into naive T (CD45RO^−^CD27^hi^), T_CM_ (CD45RO^+^CD27^+^), T_TM_ (CD45RO^+^CD27^int^), and three effector subtypes: T_EM_ (CD45RO^+^CD27^−^), CD45RO^−^CD27^−^ T_EMRA_ and CD45RO^−^CD27^int − lo^ T_EMRA_. **B**, Frequency of naïve, TCM, TTM, and effector T cells among CMVpp65_495 − 503_ MHC-I-tetramer^+^ T cells comparing the age groups of 20–40 and 60 + year old healthy volunteers. **C**, CMVpp65_495 − 503_ MHC-I-tetramer^+^ T effector T cells were further dissected into T_EM_, CD27^−^ T_EMRA_ and CD27^int^ T_EMRA_ groups as indicated. Mann-Whitney U test, *p < 0.05; **p < 0.01

To investigate the differentiation status of CMV-specific T cells, we used antibodies against CD45RO and CD27 (Fig. 3A). It is well-established that naive T cells highly express CD27 while lacking expression of CD45RO. CD27 expression is gradually lost when T cells differentiate into central memory (T_CM_), transitional memory (T_TM_), effector memory (T_EM_), and terminally differentiated effector cells re-expressing CD45RA (T_EMRA_ or: T_TE_ cells) [40]. Interestingly, the latter usually, but not always, lack CD27 [27, 41, 42]. In our approach, we subgrouped CD8 + T cells by applying a basic set of markers into T naive (CD45RO^−^CD27^hi^), T_CM_ (CD45RO^+^CD27^+^), T_TM_ (CD45RO^+^CD27^int^), T_EM_ (CD45RO^+^CD27^−^), as well as CD45RO^−^CD27^−^ and CD45RO^−^CD27^int − lo^ T_EMRA_ cells within the CMVpp65 MHC-I-tetramer^+^ cells and investigated the frequencies in relation to age (Fig. 3B and C). The CMVpp65-specific T_TM_ as well as the rare specific naive T cells were decreased in the elderlies compared to the young adults. On the other hand, elderlies had increased CMVpp65-tetramer^+^ cell proportion within the T_EM_ and the T_EMRA_ subtypes, while T_CM_ frequencies were not significantly different between the groups. Applying a finer categorization, the T cell subtypes found to be increased carried the marker combinations CD45RO^−^CD27^−^ and CD45RO^−^CD27^int − lo^. These differences were also visible by trend when comparing the elderly participants in age groups of five years (Supplemental Fig. 3).

### CMV-specific IgG is associated with higher frequencies of CMV-specific T_EM_ and CD45RO^−^/CD27^−^ T_EMRA_

**Fig. 4:**
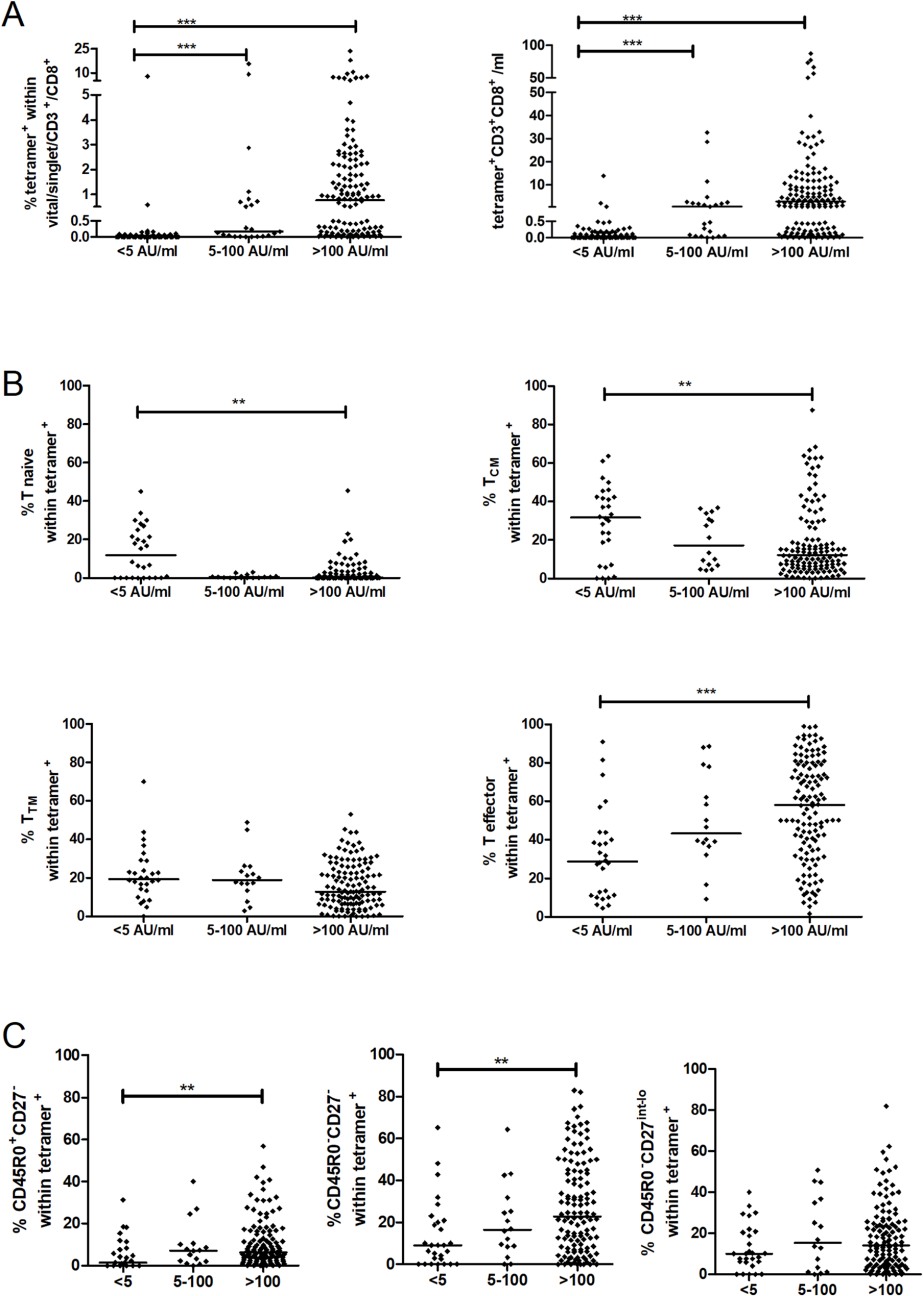
Frequency and differentiation status of CMV-specific T cells with regard to anti-CMV-IgG titers. Healthy volunteers were grouped according to the serum titers of anti-CMV-IgG (AU/ml) as indicated. **A**, Frequency and absolute counts of CMVpp65_495 − 503_ MHC-I-tetramer^+^ T cells. **B**, Frequency of naïve, T_CM_, T_TM_, and effector T cells among CMVpp65_495 − 503_ MHC-I-tetramer^+^ T cells. **C**, CMVpp65_495 − 503_ MHC-I-tetramer^+^ T effector T cells were further dissected into T_EM_, CD27^−^ T_EMRA_ and CD27^int^ T_EMRA_ groups as indicated. Kruskal-Wallis test with Dunn´s multiple comparison testing, *p < 0.05; **p < 0.01; ***p < 0.001

We next assessed whether the subtypes of CMV-specific T cells were associated with the CMV-specific antibody response. The humoral response to CMV was measured using clinical routine serum analytics during sample collection as reported earlier [35]. Higher frequencies and higher absolute counts of CMVpp65-specific T cells were detectable in donors with high antibody titers (Fig. 4A). Dissecting these T cells regarding their differentiation status, we found lower CMV-IgG levels to be associated with the percentage of naive and T_CM_ CMV-specific T cells (Fig. 4B). On the other hand, both the T_EM_ and CD45RO^−^/CD27^−^ T_EMRA_ CMV-specific T cells were detected more frequently in donors with higher specific antibody levels (Fig. 4C). These data indicate overall stronger adaptive immune responses to CMV infection in certain individuals, which did not affect immune response to other latent viruses, such as Herpes simplex virus (HSV) and Varicella zoster virus (VZV) (Supplemental Fig. 4).

### Systolic blood pressure is associated by trend with higher frequencies of CMV-specific CD45RO^−^/CD27^−^ T_EMRA_

**Fig. 5:**
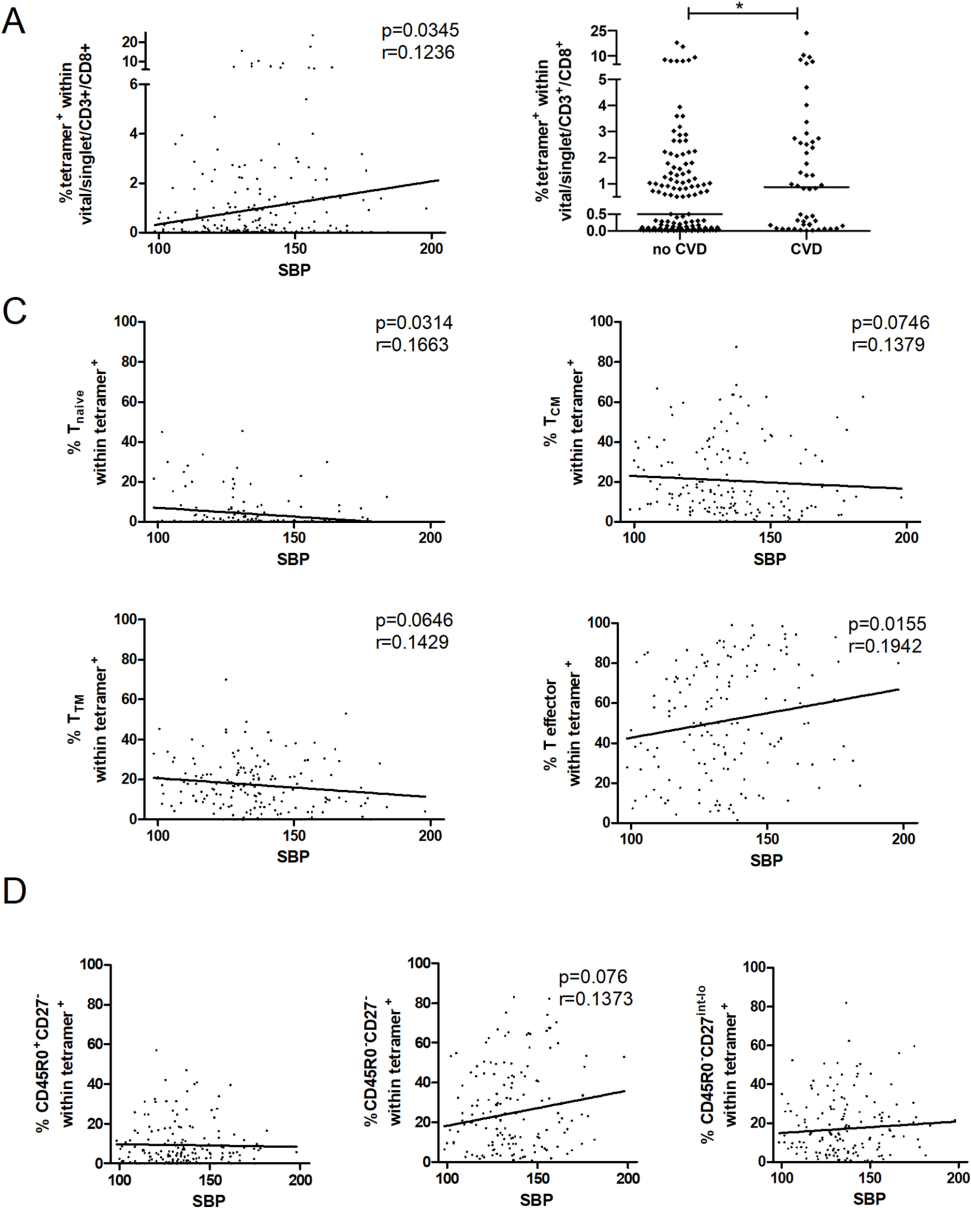
Association of the frequency of CMV-specific T cells and systolic blood pressure. **A**, Frequency of CMVpp65_495 − 503_ MHC-I-tetramer^+^ T cells plotted against systolic blood pressure (SBT). **B**, Frequency of CMVpp65_495 − 503_ MHC-I-tetramer^+^ T cells comparing individuals with or without a self-reported history of cardiovascular diseases (CVD), i.e. myocardial infarction, angina pectoris, heart failure, cardiac arrhythmias or peripheral artery disease. Mann-Whitney U test, *p < 0.05. **C**, Frequency of naïve, T_CM_, T_TM_, and effector T cells among CMVpp65_495 − 503_ MHC-I-tetramer^+^ T cells plotted against SBP. **D**, CMVpp65_495 − 503_ MHC-I-tetramer^+^ T effector T cells were further dissected into T_EM_, CD27^−^ T_EMRA_ and CD27^int^ T_EMRA_ groups as indicated. Spearman’s rank correlation, Spearman´s r and p as indicated where applicable

Adding to previous reports on the association of CMV-positivity and risk factors for cardiovascular diseases, we investigated a connection to specific T cells and their differentiation status. 50 participants reported a history of cardiovascular diseases (CVD) (Supplemental Table 1), including myocardial infarction, angina pectoris, heart failure, cardiac arrhythmias and peripheral artery disease. These donors had significantly higher frequencies of CMVpp65-specific CD8^+^ T cells comparing to participants without cardiovascular problems, and further, systolic blood pressure (SBP) was associated with the overall frequency of these cells (Fig. 5A, B). While SBP was associated with the absolute count of CMVpp65-specific CD8^+^ T cells as well (*p* = 0.044; Spearman´s *r* = 0.117), no association between the CMV-specific T cell frequency and the diastolic blood pressure were detectable (data not shown). We also did not detect any differences in CMV-IgG titers with regard to CVD (Supplemental Fig. 5). Broken down into the subtypes, the correlation of SBT with effector T cells was stronger compared to the overall CMVpp65-specific CD8^+^ T cells, while the naive, T_CM_ and T_TM_ subtypes followed the opposite trend (Fig. 5C). Dissecting the effector T further, no significant correlation was observable, although it appears as if mostly the CD45RO^−^CD27^−^ T cells, but not the CD45RO^+^CD27^−^, were increased with higher SBP (Fig. 5D). Of note, SBP was also positively correlated with age (Supplemental Fig. 6), however, age was associated with the CD45RO^+^CD27^−^ but not the CD45RO^−^CD27^−^ T cells in our hands (Fig. 3C).

## Discussion

CMV infection currently affects approximately 56% of people in Germany [43], while in other parts of the world often higher rates are observed [44]. It has been described as one of the most influential non-genetic impacts on the immune system [6], since the reshaping the cellular composition is impacting vaccinations and the immune response to infections [39]. Many population or cohort studies rely on the CMV-IgG serostatus to investigate the impact of CMV on the immune response, and the RESIST senior individuals cohort has been analyzed in this regard in an earlier study [45].

This study used the specific CD8^+^ T cell response as a basis for investigating the remodeling of human immunity by CMV. Thereby we can confirm here most of the earlier findings, including that chronic encounter of the virus leads to accumulation of late-differentiated CD8^+^ T cells. The increase in the late-stage effector lymphocyte populations are usually seen as a result of the continuous attempts of the immune system to control CMV reactivation and are part of the success of this operation [46, 47]. In our hands, these changes were that strong that differences were detectable on the level of leukocyte counts in our cohort: total CD8^+^ as well as CD4^+^CD8^+^ double-positive T cell frequencies and absolute counts were increased in individuals with a strong cellular immune response to CMV, independent of age. Earlier studies have shed light on the phenotype of CD4^+^CD8^+^ double-positive T cells, which are often found in patients with chronic virus infections, and described Th1/Tc2 cells, with enhanced cytokine expression, proliferation and cytotoxic activity [48–51].

This project applied MHC tetramers, which carried the well-known and widely reactive CMV epitope NLVPMVATV. MHC multimer staining has certain advantages over cell stimulation assays, such as i, not being influenced by proliferation capacity, ii, not being influenced by cytokine secretion capacity, and iii, not being influenced by bystander cells. On the other hand, cell stimulation and read-outs such as proliferation or cytokine expression allow to investigate the immune response without restriction to single epitopes. E.g., a study that investigated elderly CMV-seropositive individuals by peptide stimulation detected a positive association between CMV-serostatus and frequencies of T_EMRA_ cells among CMV-reactive CD8 + T cells, while naive, central and effector memory cells were negatively associated with CMV serostatus [15]. These findings are generally in line with our findings, despite slight differences in defining T cells subtypes. However, we did not observe significant sex-related differences with regard to CMV p65-specific T cell subtypes. This matches former studies, which compared peptides from different CMV antigens in this respect [52]. In that study, sex-related differences were restricted to other antigens aside from UL83 (pp65).

Previous studies addressed the number, cytokine production, and growth potential of CMVpp65_495 − 503_-specific T cells, pointing out a T_EMRA_ phenotype with reduced proliferative capacity [53]. Adding to that, our study shows that different subsets of effector cells are associated with age, seropositivity, and SBT. Especially the CD45RO^+^CD27^−^ T_EM_ and CD45RO^−^CD27^int − lo^ T_EMRA_ fractions of the CD8^+^ T effector cells were increased with age. On the other hand, CMV-seropositivity was merely associated with CD45RO^+^CD27^−^ T_EM_ and CD45RO^−^CD27^−^ T_EMRA_ frequencies, which was mirrored by increased SBT. This indicates that not age or CMV-infection per se renders elderly CMV-positive individuals susceptible to CVD, but the cellular immune response to CMV.

While studies have reported, that a strong response to CMV was associated with longer lifespan [54], the continuous combating the CMV attempts to reactivate is nevertheless associated with certain costs for the organism. The iAge score, which was developed as a predictive score for inflammaging and was shown to be able to predict multimorbidity, correlated with CMV positivity in a multiple regression model [55]. Elderly individuals seropositive for CMV or with clonal expansion of T_EMRA_ cells were reported to show poor humoral responsiveness following influenza vaccination [7, 56–58]. Further, the specific T-cellular response to another herpesvirus, EBV, was reduced in CMV-seropositive compared to seronegative elderly (but not young) individuals [59]. The observed increased risk of seropositive individuals to develop CVD decades ago [12] has been challenged by subsequent studies: Kirkham et al. found a positive association of central aortic (carotid-to-femoral) pulse wave velocity (PWV) with CMV serostatus and memory CD4 T cell frequency only in men but not women [22]. In combined multiple observational cohort studies in healthy older adults, Chen et al. found no correlation between CMV seropositivity and all-cause or cardiovascular mortality [23]. Similarly, large population-based prospective cohort study of middle-aged, largely healthy adults in UK also found no significant association between CMV seropositivity and risk of CVD [24].

It has been hypothesized that the effect of CMV on arterial stiffness and CVD is associated with reactivation of CMV in endothelial cells and the subsequent T-cell response. On the cellular side, it was shown that expression of the chemokine receptor CX3CR1 may target CMV-specific CD8+ [60] and CD4 + T cells [61] to the endothelium, a target tissue of CMV infection, to locally induce vascular inflammation. Bolovan-Fritts et al. showed that the cytokine response by cytomegalovirus-reactive CD4 + T cells induce fractalkine expression on endothelial cells [62], which is chemotactically attracting CX3CR1 + cells such as NK or T cells, presumably leading to endothelial damage. However, no association of CMV-specific CD4 + T cells with central aortic pulse wave velocity was detectable in a recent study [22]. Focusing CD8 + T cells, pp65 is expressed during reactivation and not during latency, and therefore a suitable target in the scenario, compared to other frequently analyzed CMV antigens such as IE1.

Recent studies observed that frequencies of circulating CMV-specific CD8^+^ T cells are associated with the PWV as a marker for arterial stiffness [9]. In that study, which relied on 415 bona fide seropositive participants, CMV-specific T cells were defined by immunostaining of IFN-g, TNF-a, or CD107 after stimulation with pp65 peptides. The study describes correlations for each of the three immunostainings with PWV, but no correlation with the humoral response to CMV, and therefore conclude that the specific T cell response is of more importance than the infection itself. Analyzing 207 patients with hypertension, a correlation of arterial stiffness with CMV pp65-specific CD8^+^ T-cell responses could be observed [10]. In our hands it became apparent, that especially the specific T effector-like T cell subset and further the CD45RO^−^CD27^−^ T_EMRA_ cells were increased with SBP. These data suggest that the differentiation status of CD8 + CMV-specific T cells influences the cardiovascular system and can serve as a predictor for disease. While aging itself represents a risk factor for hypertension, and overall CD8^+^CD27^−^ T cell frequencies have been reported to predict for this [42], we observed that different subtypes of CMV-specific T cells are associated with aging and SBT, respectively. The detection of the chemokine receptor CX3CR1 as well as further markers of immunosenescence, such as CD57, KLRG1, or SLAMF7 [27, 41, 63] would shed further light on the nature of these cells.

It should be noted that single donors may have been subjected to HLA-A*02:01 tetramer-staining, who carried other HLA-A2 alleles, which may have led to false negative results. However, these alleles are comparably rare, and this approach had the advantage of a quick turnover and allowed analyzing the CMV-specific T cells directly without further freezing and storage. As mentioned, this study was based on the hypothesis that the effect of CMV on atherosclerosis and CVD is associated with the reactivation of CMV in endothelial cells and the subsequent T-cell response. However, there is also the possibility that CVD itself may have an impact on CMV reactivation in a vicious circle.

In conclusion, we made use of the RESIST SI cohort, which resembles the aging population in Germany [35], to investigate associations of the specific cellular immune response to CMV with aging and comorbidities. While confirming previous findings, we show that aging is associated with a different phenotype of CMV-specific T cell response compared to the humoral response and increased SBP. Analysis of specific T cell subtypes may therefore help to predict cardiovascular anormalities.

## Electronic supplementary material

Below is the link to the electronic supplementary material.

Supplementary Material 1

## Data Availability

The datasets used and analysed during the current study are available from the corresponding author on reasonable request.
