# Supplementary material for: Different CMV-specific effector T cell subtypes are associated with age, CMV serostatus, and increased systolic blood pressure
Source: Immun Ageing. 2025 Jul 3;22:27. doi: 10.1186/s12979-025-00523-x (PMC12224750; doi:10.1186/s12979-025-00523-x)
Supplement: Supplementary file 1 — Supplementary Material 1 [file 12979_2025_523_MOESM1_ESM.docx]

**Supplemental Data**

Supplemental Table 1. **Participants who reported a history of cardiovascular diseases (CVD).**

| **No** | **Cardiac arrhythmias** | **Mycardial infarcation** | **Angina pectoris** | **Heart failure** | **Peripheral artery disease** |
| --- | --- | --- | --- | --- | --- |
| 1 | 0 | 0 | 1 | 0 | 0 |
| 2 | 1 | 0 | 1 | 0 | 0 |
| 3 | 0 | 0 | 1 | 0 | 0 |
| 4 | 0 | 0 | 0 | 0 | 1 |
| 5 | 0 | 0 | 1 | 0 | 0 |
| 6 | 1 | 1 | 0 | 0 | 0 |
| 7 | 0 | 0 | 1 | 0 | 1 |
| 8 | 0 | 0 | 1 | 0 | 0 |
| 9 | 1 | 0 | 0 | 0 | 0 |
| 10 | 1 | 0 | 0 | 0 | 0 |
| 11 | 1 | 0 | 0 | 0 | 0 |
| 12 | 1 | 0 | 0 | 0 | 0 |
| 13 | 1 | 0 | 1 | 0 | 0 |
| 14 | 0 | 0 | 1 | 0 | 0 |
| 15 | 0 | 0 | 0 | 0 | 1 |
| 16 | 0 | 0 | 0 | 1 | 0 |
| 17 | 0 | 1 | 1 | 0 | 0 |
| 18 | 0 | 0 | 0 | 1 | 0 |
| 19 | 1 | 0 | 1 | 1 | 0 |
| 20 | 1 | 1 | 1 | 0 | 0 |
| 21 | 1 | 0 | 0 | 0 | 0 |
| 22 | 1 | 0 | 0 | 0 | 0 |
| 23 | 1 | 0 | 0 | 0 | 0 |
| 24 | 0 | 1 | 0 | 0 | 0 |
| 25 | 0 | 0 | 0 | 0 | 1 |
| 26 | 0 | 0 | 1 | 0 | 0 |
| 27 | 1 | 0 | 0 | 0 | 0 |
| 28 | 1 | 0 | 0 | 0 | 1 |
| 29 | 1 | 1 | 1 | 1 | 0 |
| 30 | 1 | 0 | 0 | 0 | 0 |
| 31 | 1 | 0 | 0 | 0 | 0 |
| 32 | 1 | 0 | 0 | 0 | 0 |
| 33 | 0 | 0 | 0 | 0 | 1 |
| 34 | 1 | 0 | 0 | 0 | 0 |
| 35 | 0 | 0 | 0 | 1 | 0 |
| 36 | 0 | 0 | 0 | 1 | 0 |
| 37 | 1 | 0 | 0 | 0 | 0 |
| 38 | 1 | 0 | 0 | 1 | 0 |
| 39 | 1 | 0 | 0 | 1 | 0 |
| 40 | 0 | 0 | 1 | 0 | 0 |
| 41 | 0 | 0 | 0 | 1 | 0 |
| 42 | 0 | 0 | 1 | 0 | 0 |
| 43 | 1 | 0 | 1 | 0 | 0 |
| 44 | 1 | 0 | 0 | 1 | 0 |
| 45 | 1 | 0 | 0 | 0 | 0 |
| 46 | 0 | 0 | 0 | 0 | 1 |
| 47 | 1 | 0 | 0 | 0 | 0 |
| 48 | 1 | 0 | 0 | 0 | 0 |
| 49 | 0 | 1 | 0 | 0 | 0 |
| 50 | 0 | 0 | 0 | 0 | 1 |

**
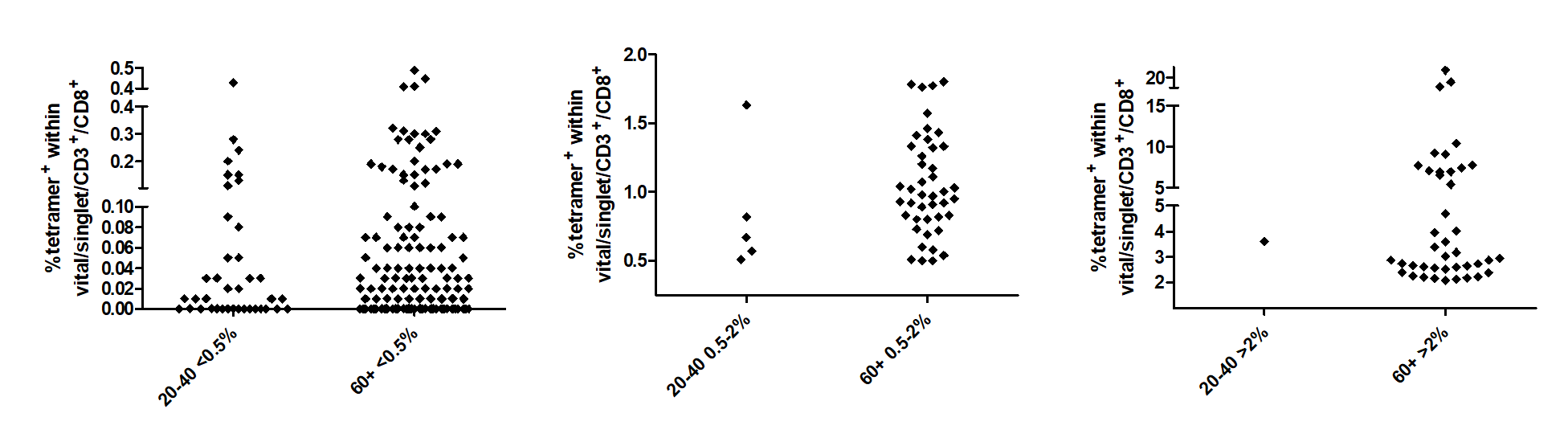
**

Supplemental Figure 1. **Frequency of CMV-specific T cells.** Frequency of CMVpp65_495-503_ MHC-I-tetramer^+^ T cells comparing the age groups of 20-40 and 60+ year old healthy volunteers. To enhance visualization, separate plots for participants with < 0.5% (left), 0.5-2% (middle), and > 2% (right) are displayed.


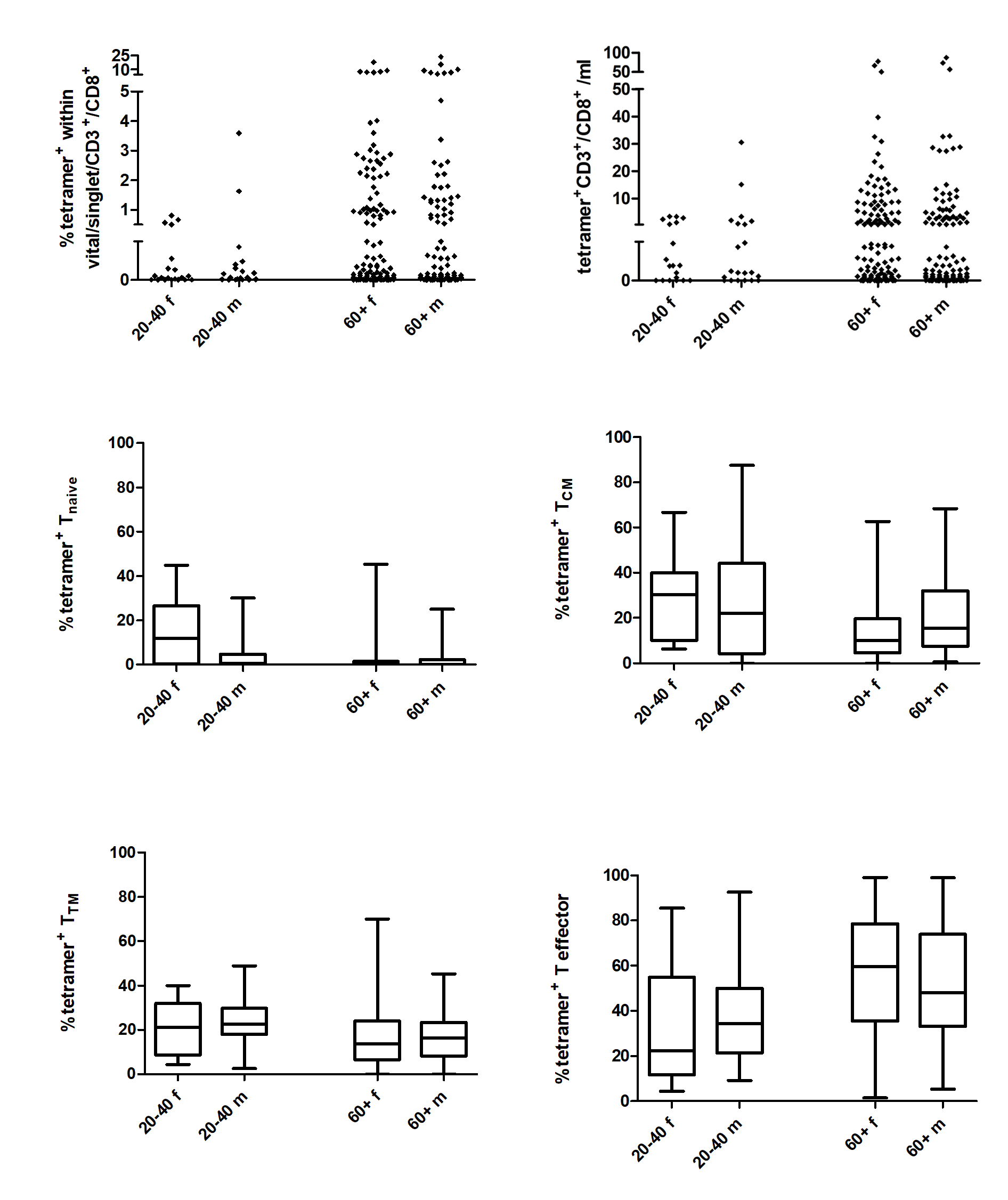


Supplemental Figure 2. **Frequency and differentiation status of CMV-specific T cells with regard to sex.** Upper: Frequency and absolute counts of CMVpp65_495-503_ MHC-I-tetramer^+^ T cells in healthy volunteers by male and female sex. Lower: Frequency of naïve T, T_CM_, T_TM_, and effector T cells among CMVpp65_495-503_ MHC-I-tetramer^+^ T cells in healthy volunteers by male and female sex.


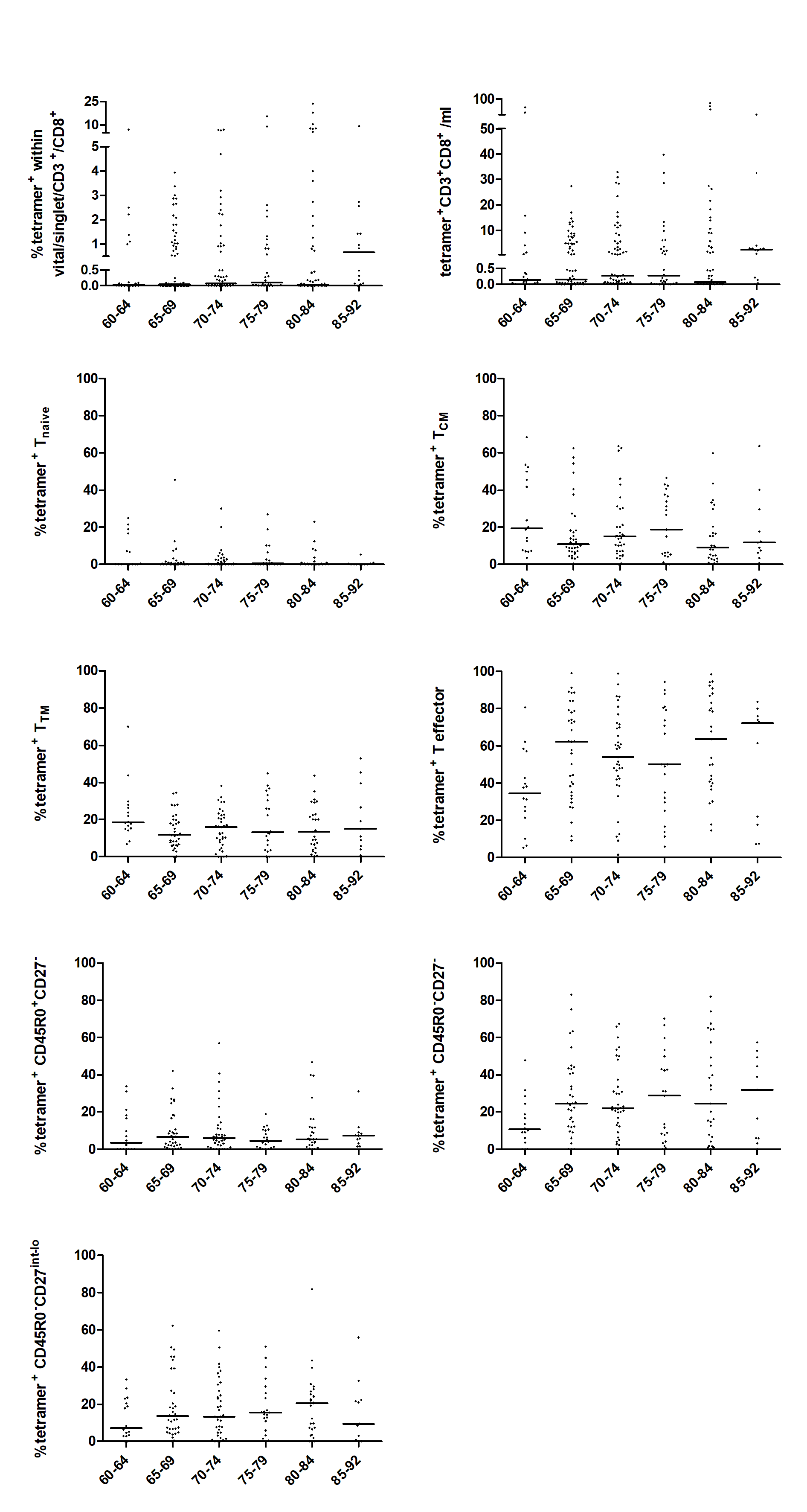


Supplemental Figure 3. **Frequency and absolute counts of naïve, TCM, TTM, and effector T cells among CMVpp65_495-503_ MHC-I-tetramer^+^ T cells among the 60+ year old healthy volunteers, dissected into groups of 5 years of age.** CMVpp65_495-503_ MHC-I-tetramer^+^ T effector T cells were further dissected into T_EM_, CD27^-^ T_EMRA_ and CD27^int^ T_EMRA_ groups as indicated.


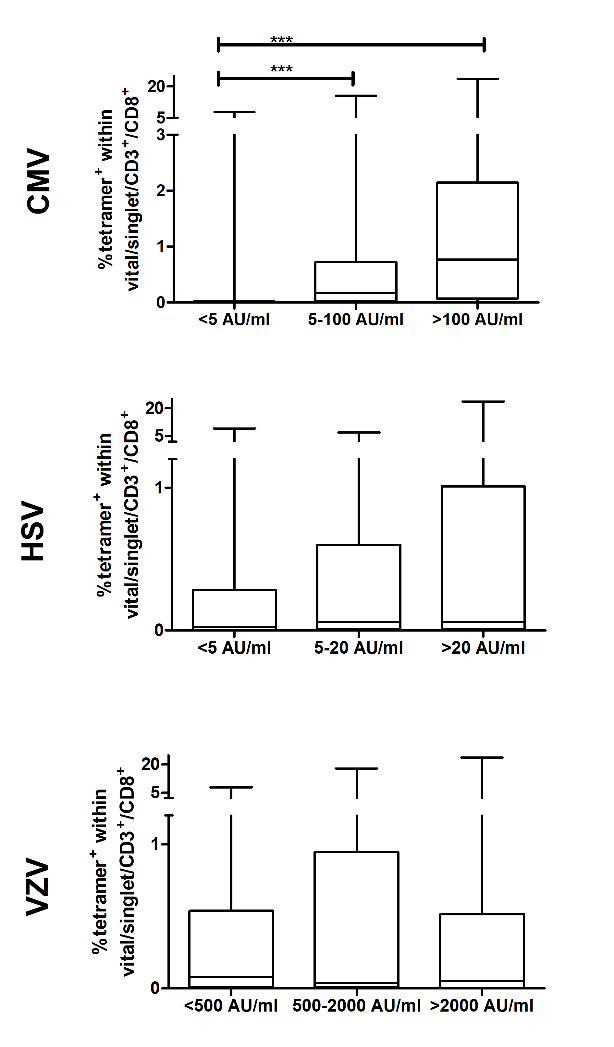


Supplemental Figure 4. **Frequency of CMV-specific T cells with regard to anti-herpesvirus IgG titers.** Frequency of CMVpp65_495-503_ MHC-I-tetramer^+^ T cells compared among individuals with low, intermediated, and high serum titers against CMV, HSV, and VZV as indicated. Kruskal-Wallis test with Dunn´s multiple comparison test, ***p < 0.001.


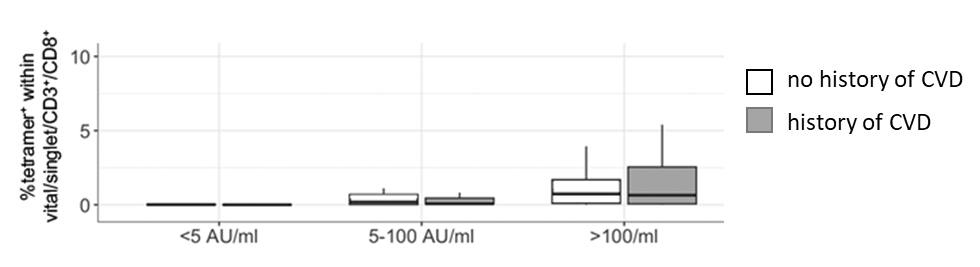


Supplemental Figure 5. Frequency of CMVpp65_495-503_ MHC-I-tetramer^+^ T cells with regard to CMV IgG titers (AU/ml) comparing individuals with (grey) or without (white) a self-reported history of cardiovascular diseases (CVD), i.e. myocardial infarction, angina pectoris, heart failure, cardiac arrhythmias or peripheral artery disease.


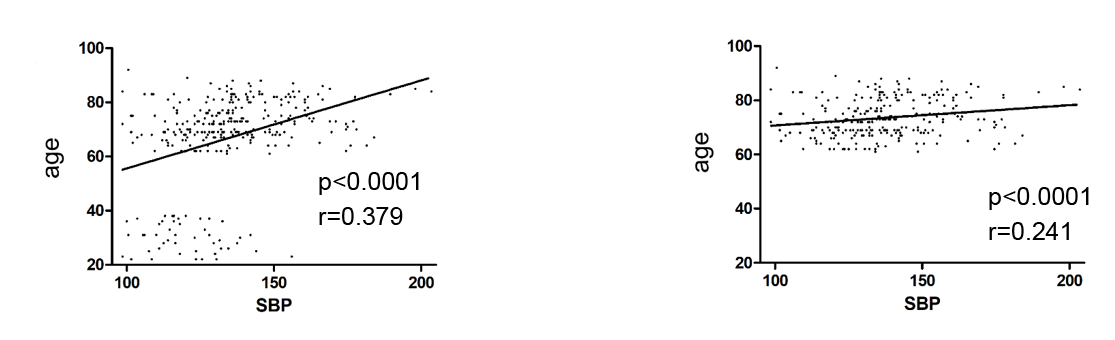
Supplemental Figure 6. **Correlation analysis of age and systolic blood pressure** (SBP). Left, including 20-40 year-olds and 60+ year-olds. Right, Separate analysis of 60-year-olds. Spearman's rank correlation, Spearman´s r and p as indicated.
